# Exploring Mobile EMA and GPS to Understand Social Connectedness in Community-Dwelling Older Adults

**DOI:** 10.1093/geroni/igaf122.755

**Published:** 2025-12-31

**Authors:** JiYeon Choi, Bomgyeol Kim, Seongmi Choi, JaeWon Hyun, Hun Kang

**Affiliations:** Yonsei University, Seoul, Republic of Korea; Yonsei University College of Nursing, Seoul, Republic of Korea; Daegu Catholic University, Daegu, Republic of Korea; Yonsei University, Seoul, Republic of Korea

## Abstract

Social connectedness is a key determinant of health and well-being in older adults. Ecological Momentary Assessment (EMA) has been widely used to study dynamic social interactions in daily life, and technological advancements continue to refine EMA for real-world applications. This presentation shares our experience of conducting a series of studies that assess social connectedness and associated psychosocial responses among community-dwelling older adults using mobile EMA and Global Positioning System (GPS) tracking. In 2023, in collaboration with the Active Senior Living Lab Support Group (Seongnam Senior Industry Innovation Center, Gyeonggi-do, South Korea), we established a cohort of older adults (n = 35, 72.2±6.7 years old) for longitudinal research on social connectedness. A pilot study assessed the feasibility of mobile EMA-based data collection by measuring social activities and emotions four times daily for two weeks (56 data points). Thirty-four participants successfully completed data collection (completion rate, 99.7%). Four group interviews with 20 pilot study participants provided feedback on EMA applicability, assessment appropriateness, and research staff interactions. Participants reported increased awareness of their social interactions, reinforced their sense of responsibility, and fostered a stronger sense of community belonging. Based on these findings, our 2025 follow-up study integrates the use of mobile EMA with GPS tracking system to further explore the spatial dynamics of social connectedness in older adults. This approach records the location-based social engagement pattern information, thereby providing contextual insights into social connectedness, loneliness, and emotions. These findings will help to refine methodologies for assessing social connectedness and contextual factors in community-dwelling older adults.

